# Recent Progress in Quantitatively Monitoring Vesicular Neurotransmitter Release and Storage With Micro/Nanoelectrodes

**DOI:** 10.3389/fchem.2020.591311

**Published:** 2021-01-11

**Authors:** Yuying Liu, Jinchang Du, Mengying Wang, Jing Zhang, Chunlan Liu, Xianchan Li

**Affiliations:** Center for Imaging and Systems Biology, College of Life and Environmental Sciences, Minzu University of China, Beijing, China

**Keywords:** vesicle impact electrochemical cytometry (VIEC), amperometry, electrodes, neurotransmitter, exocytosis, vesicle

## Abstract

Exocytosis is one of the essential steps for chemical signal transmission between neurons. In this process, vesicles dock and fuse with the plasma membrane and release the stored neurotransmitters through fusion pores into the extracellular space, and all of these steps are governed with various molecules, such as proteins, ions, and even lipids. Quantitatively monitoring vesicular neurotransmitter release in exocytosis and initial neurotransmitter storage in individual vesicles is significant for the study of chemical signal transmission of the central nervous system (CNS) and neurological diseases. Electrochemistry with micro/nanoelectrodes exhibits great spatial–temporal resolution and high sensitivity. It can be used to examine the exocytotic kinetics from the aspect of neurotransmitters and quantify the neurotransmitter storage in individual vesicles. In this review, we first introduce the recent advances of single-cell amperometry (SCA) and the nanoscale interface between two immiscible electrolyte solutions (nanoITIES), which can monitor the quantity and release the kinetics of electrochemically and non-electrochemically active neurotransmitters, respectively. Then, the development and application of the vesicle impact electrochemical cytometry (VIEC) and intracellular vesicle impact electrochemical cytometry (IVIEC) and their combination with other advanced techniques can further explain the mechanism of neurotransmitter storage in vesicles before exocytosis. It has been proved that these electrochemical techniques have great potential in the field of neuroscience.

## Introduction

The neuron is an important structural and functional unit of the central nervous system (CNS), which controls higher mental functions. Exocytosis is a fundamental process of chemical signal transmission between neurons and the key of intercellular communication (Meldolesi and Ceccarelli, 1981). Upon stimulation at the presynaptic membrane, depolarization of the cell membrane induces the Ca^2+^ channel to open and the Ca^2+^ influx triggers the exocytosis. The vesicles then fuse with the plasma membrane and release the loaded neurotransmitters, such as catecholamines, acetylcholine (ACh), and glutamate, into the synaptic space. When released neurotransmitters bind to the specific receptors on the postsynaptic membrane, chemical signal transduction is completed. Hence, the vesicle is an essential cell organelle responsible for neuronal chemical communication. Understanding neurotransmitter storage in vesicles and the dynamic process of its exocytotic release from vesicles is of great significance in neuroscience.

Various analytical techniques, including fluorescence, electrochemistry, and mass spectrometry imaging (MSI), have been used to study the mechanism of exocytosis. Taking advantage of its great spatial resolution, fluorescence, especially total internal reflection fluorescence microscopy (TIRFM), has been successfully used to study the distribution of vesicles in a single cell and the mobility of vesicles during exocytosis (Yuan et al., 2015; Liu et al., 2017). MSI shows specific advantages in identifying the molecular information and visualizing the distribution of neurotransmitters in vesicles *in situ* (Lovric et al., 2017; Phan et al., 2017). Electrochemistry benefiting from its ultra-high temporal resolution and quantitative capability has been proven to be a powerful technique in the study of vesicle neurotransmitter release and storage.

Single-cell amperometry (SCA) is a long-standing method for studying exocytosis (Leszczyszyn et al., 1990; Wightman et al., 1991; Majdi et al., 2015; Li X. et al., 2016b,c; Ren et al., 2017). By using micro/nanoelectrodes, the electrochemically active neurotransmitters secreted from single vesicles during exocytosis, such as dopamine (DA), epinephrine, and norepinephrine, can be directly detected at a single-cell level. Through analyzing the recorded current transients caused by the oxidation of neurotransmitters under the constant potential applied on the electrode surface, one can quantify the amount of neurotransmitters released in a single exocytotic event and obtain detailed information about the exocytotic kinetics. To overcome the disadvantage of SCA that it can hardly monitor the non-electrochemically active neurotransmitters so far, nanoelectrodes equipped with the interface between two immiscible electrolyte solutions (nanoITIES) have been developed to monitor the concentration and kinetics of cholinergic transmitters released from single neurons (Shen et al., 2018; Welle et al., 2018).

With the aim to quantify the ratio of neurotransmitter release and infer whether the contents of vesicles are completely released during exocytosis, it is necessary to examine the storage of neurotransmitters in individual vesicles. Ewing's group developed vesicle impact electrochemical cytometry (VIEC) and intracellular vesicle impact electrochemical cytometry (IVIEC) which can detect the neurotransmitter content of isolated vesicles and vesicles in living cells, respectively. In VIEC, the microelectrode was placed directly into the vesicle suspension prepared from cells, and vesicles would be adsorbed on the electrode surface. Under a constant potential, the vesicles on the surface of the electrode rupture by electroporation and then their content are expelled and thus quantified on the electrode. The principle of IVIEC is similar to that of VIEC with its own advantage of monitoring vesicular content in living cells. The combination of SCA, VIEC/IVIEC, and other advanced analytical techniques provides efficient methodologies for studying exocytosis, vesicle properties, neuro-pharmacology, and neuro-toxicology.

This review aims to report new developed approaches, including SCA, ITIES, VIEC, and IVIEC, and their advanced progress in quantitatively monitoring vesicular neurotransmitter release in exocytosis and initial neurotransmitter storage in individual vesicles with electrochemistry. We have made an attempt to be comprehensive, but this review focuses on the last 5 years.

## Single-Cell Amperometry for Monitoring Neurotransmitter Release During Exocytosis

Single-cell amperometry (SCA) originated from the pioneering studies by Wightman's group (Wightman et al., 1991). In this approach, a disk carbon fiber microelectrode is placed in proximity to the nerve cells to form a semi-synapse. Once stimulated, neurotransmitters released from cells are oxidized at the electrode thus producing a current transient. The dynamic information related to the fusion pore and release kinetics can be obtained by analyzing the current transients. The innovative development of SCA has initiated a new era in the neuroscience benefiting from its ultra-high temporal resolution (Majdi et al., 2015; Raghupathi et al., 2016; Moreira et al., 2017; Gu et al., 2020; He and Ewing, 2020). It has not only been widely used to monitor the exocytotic process of nerve cells but also been used to study the effects of various drugs on the exocytosis process (Machado et al., 2002; Manning et al., 2012; Trouillon and Ewing, 2013, 2014; Taleat et al., 2019; Ren et al., 2020).

### Catecholamines

**Figure 1:**
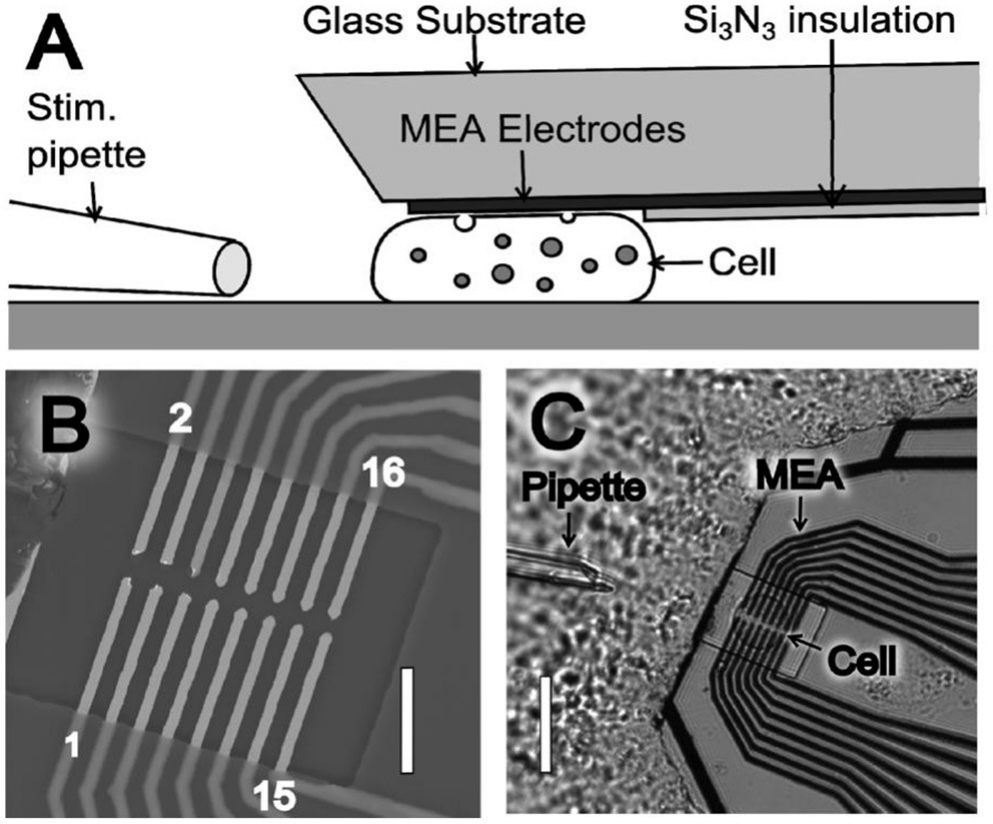
**(A)** Illustration of the microelectrode array (MEA) device side view (not drawn to scale), where the thin film platinum electrodes are placed to touch and probe a cell. **(B)** Scanning electron microscope (SEM) image of a MEA showing the 16 exposed microelectrodes within an open window of the Si_3_N_3_ insulation layer. MEA electrodes are indicated by odd numbering (1–15) in the lower electrode row and even numbering (2–16) in the upper electrode row. Scale bar is 10 μm. **(C)** Light microscopy image of a MEA probe placed on top of a chromaffin. Scale bar is 30 μm. Reprinted from Wigstrom et al. (2016), with permission from American Chemical Society.

For the last three decades, improving the spatial resolution and readout speed of SCA are two of the most important areas for its application in neuroscience studies. One main strategy for this purpose is microelectrode arrays (MEAs), which decreases the spatial resolution down to ~1 μm along with multiple readouts at different sites simultaneously on a single cell. Usually, cells are randomly attached on MEAs fabricated with lithography, leading to high chance that cells are not placed at the ideal site (Picollo et al., 2016; Huang et al., 2018; Tomagra et al., 2019). Recently, Wigstrom et al. developed a movable 16-platinum MEA, which is capable of being placed in close proximity to the membrane of single attached chromaffin cells (Wigstrom et al., 2016). As shown in Figure 1, this MEA could be placed on top of a cell by accurate placement with mechanical control, thus allowing multiple recordings at different cells cultured in the same batch with the same MEAs. Moreover, if coupled with the regular bottom MEAs, this on-top MEA can greatly enlarge the cell surface area imaged with electrochemistry with a comparable spatial resolution to optical methods, but with a much higher temporal resolution.

Another approach to improve the spatial resolution of SCA is decreasing the electrode size. In the last 5 years, the development of nanoelectrodes has opened a new window for SCA to monitor the catecholamine release during exocytosis with a higher spatial resolution. For instance, a new kind of Au disk nanoelectrode with a radius down to 3 nm was developed and used for monitoring DA release from single rat pheochromocytoma (PC12) cells by using SCA (Liu et al., 2015). The results showed that the electrode could record eight current responses corresponding to DA release from eight vesicles in the active region, which proved its ability to identify characteristics of different release sites in the same region. Moreover, this nanoelectrode could define different release sites in the same active zone on a PC12 cell owing to its extremely high spatial resolution. The preparation method for this nanoelectrode is relatively practical and can be extended to other materials, leading to great potential for single-cell research.

**Figure 2:**
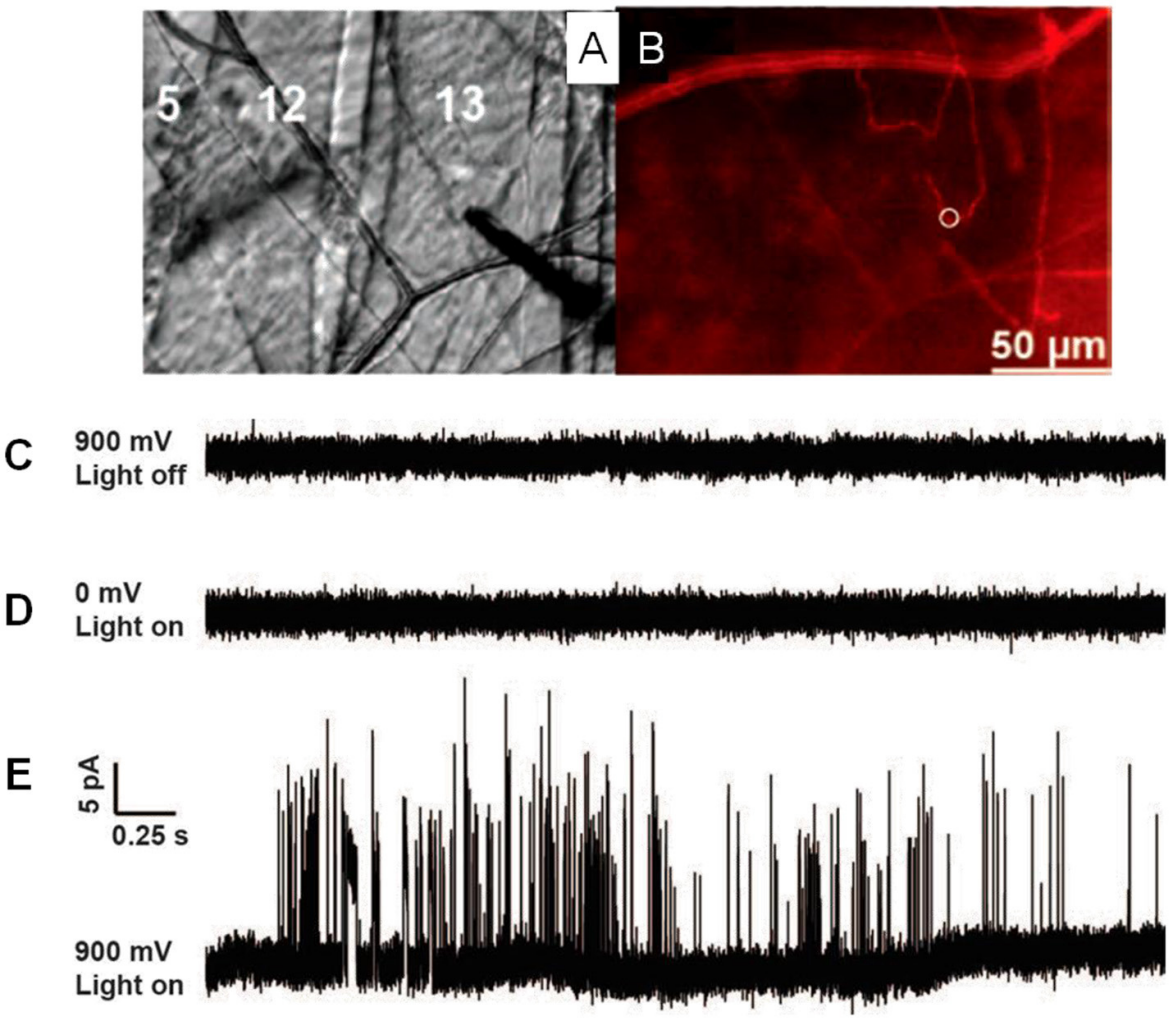
**(A)** Microelectrode placement on the Type II varicosities in muscle 13 in *Drosophila* larvae. **(B)** Same view as 1C but with fluorescence, *m*Cherry-labeled octopaminergic terminals (Type II varicosities) presented as a red line and the white ring shows the placement of the microelectrode. **(C)** A potential of 900 mV (vs Ag/AgCl reference electrode) was applied, with no light stimulation. **(D)** A potential of 0 mV was applied, with blue light stimulation. **(E)** A 900-mV potential, stimulated with blue light. Same scale for all traces. Reprinted from Majdi et al. (2015), with permission from Wiley Online Library.

One breakthrough of the application of SCA in neuroscience in the last 5 years is the real-time recording of catecholamine release from single exocytosis events *in vivo*. Ewing's group developed a novel method to record the neurotransmitter release from exocytosis at single varicosities in the *Drosophila* larval system with SCA (Figure 2) (Majdi et al., 2015). When they placed a regular carbon fiber microelectrode in close proximity to Type II varicosities which were stimulated with blue light, a train of current transients representing the oxidation of octopamine released were recorded at a potential of + 900 mV (vs. Ag/AgCl). Once analyzing each current transient in detail, various shapes of exocytotic events, including simple and complicated ones, reveal that partial release is prevalent in this condition.

**Figure 3:**
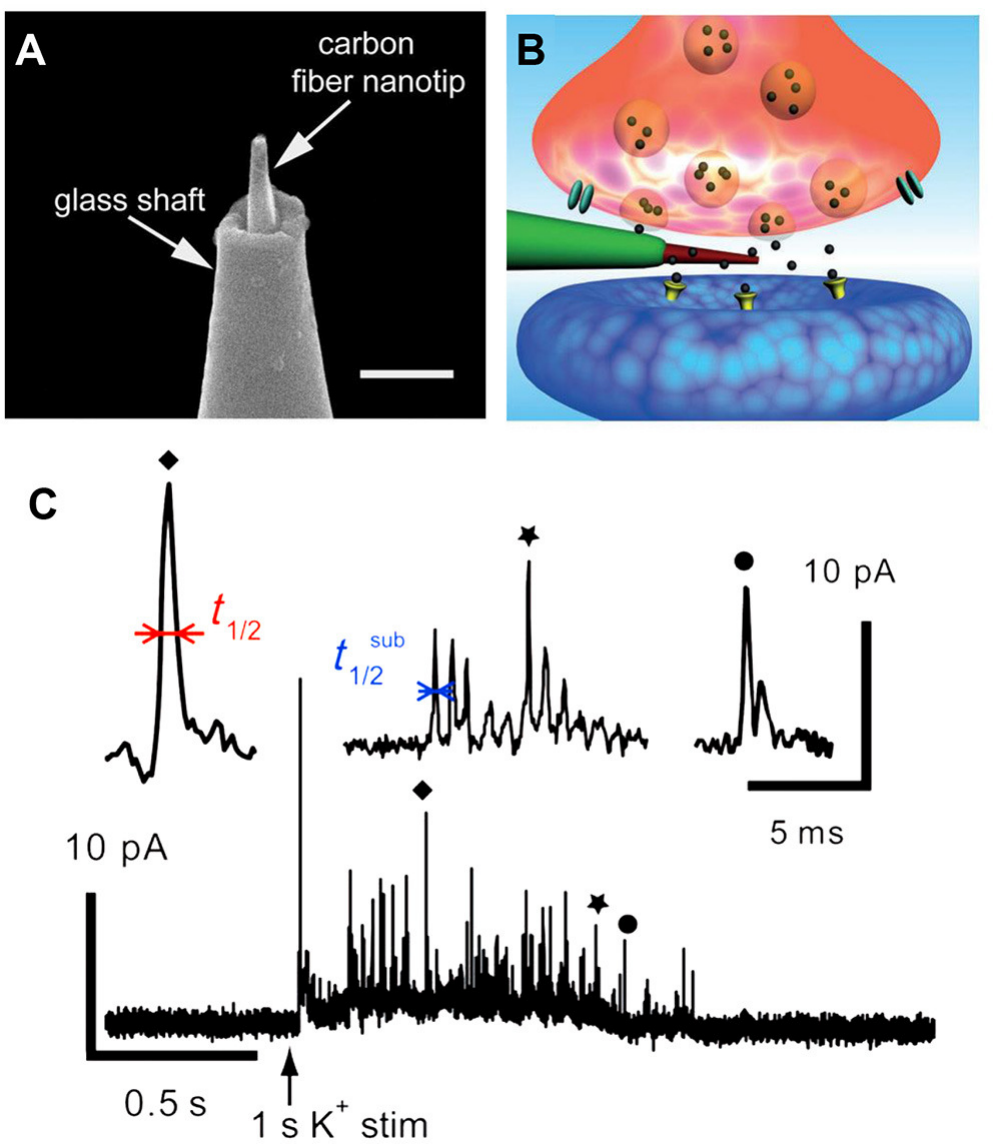
**(A)** Amplified picture of the tip of the nanoelectrode; the scale bar is 1 μm. **(B)** Schematic representation of a nanosensor's tip inside an individual synapse. **(C)** High K^+^-induced amperometric spike and two complex events were amplified above. Reprinted from Li et al. (2014), with permission from Wiley Online Library.

In another elegant study, a brand new conical carbon fiber nanoelectrode (CFNE) fabricated by flame-etching the cylindrical carbon fiber microelectrode was placed into a single trained synapse formed between cultured superior cervical ganglion (SCG) and sympathetic neurons to monitor catecholamine release during exocytosis, as displayed in Figure 3 (Li et al., 2014). When high K^+^ was applied near the synapse, typical current transients were recorded in ca. 48% of cases. Approximately 42% of spikes exhibited a single rising phase followed by a single falling one (simple events), while 58% of events consisted in sequences of multiple sub-spikes (complexed events) with overall longer durations and more released norepinephrine than those of single events, respectively.

To elucidate the nature of the vesicular exocytosis in synapse, Li et al. reconstructed *in vivo*-like oriented neuronal networks in microfluidic channels between SCG neurons and their effector smooth muscle cells (SMC), which were placed in two separate microfluidic chambers initially (Li Y. et al., 2015). In consistency with the result in a Petri dish, they also recorded both single and complex spikes. However, the probability of successful detection increased from 61.0% in a Petri dish to 74.6% in this microfluidic chip, suggesting that the microfluidic strategy worked more effectively to form *in-vivo*-like neuronal synapses. Furthermore, the postsynaptic potential was successfully monitored with a glass nanopipette electrode, which again indicated that SCG-SMC synapses built in a microchip functioned effectively. This unique approach offers a platform to understand the true nature of neuronal exocytosis and the effect of drugs or chemicals, for example, harpagide, on it (Tang et al., 2020).

### Dual Probes for DA

**Figure 4:**
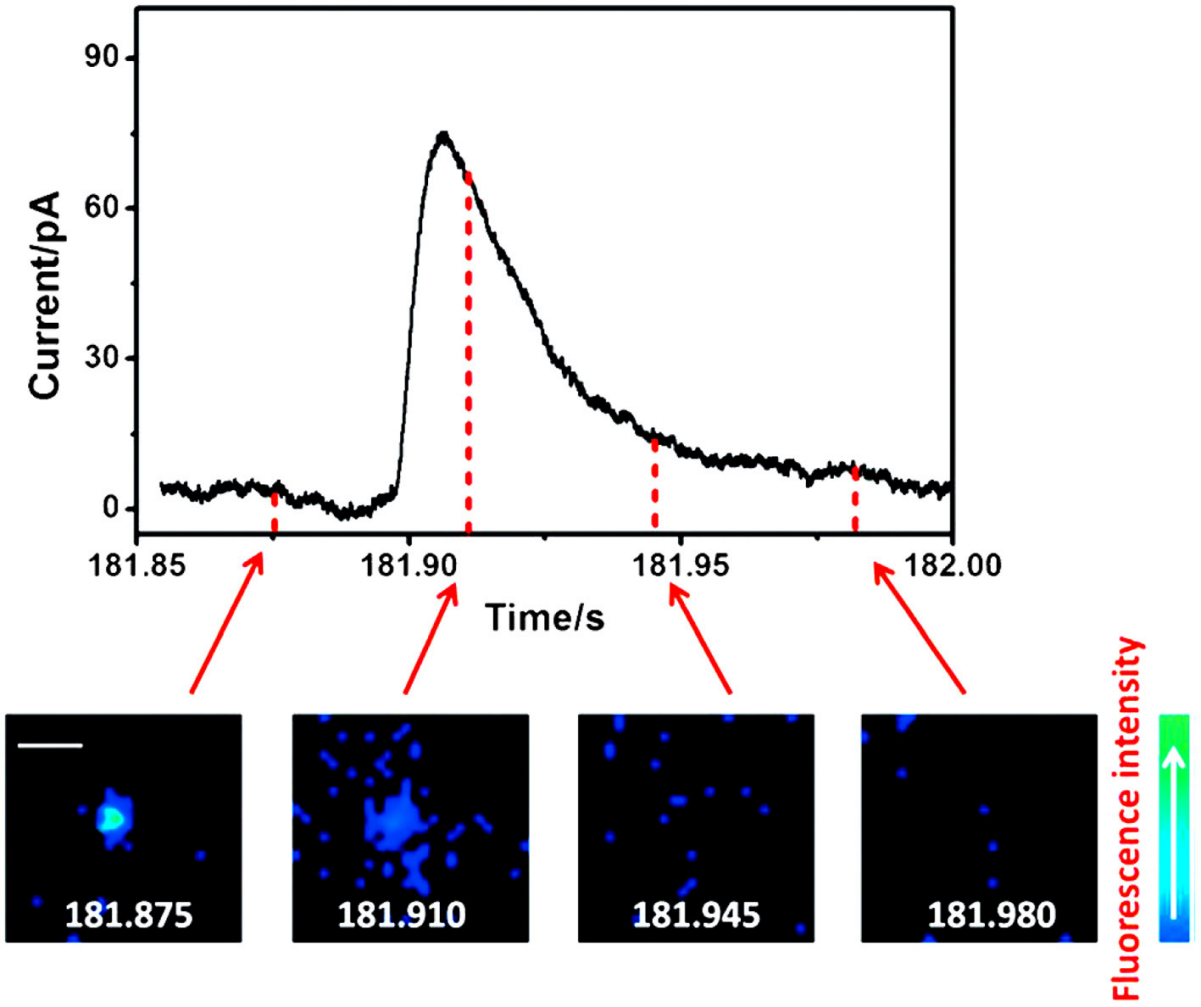
Correlation of amperometric and fluorescence information for a single exocytotic event of fluorescent false neurotransmitter102 (FFN102)-stained BON N13 cells over an indium tin oxide (ITO) microdevice. Top: An exocytotic event appeared as the current spike in electrochemical detection. Bottom: Sequential pseudocolor total internal reflection fluorescence microscopy (TIRFM) images of a single exocytotic event viewed as a flash of fluorescence. Scale bar = 500 nm. Reprinted from Liu et al. (2017), with permission from Wiley Online Library.

Fluorescence imaging, especially total internal reflection fluorescence microscopy (TIRFM), has aroused great interest in the neurotransmission study, such as exocytosis, benefiting from its great sensitivity for quantification and excellent spatial resolution capable of revealing the distribution of secretory vesicles and tracking their motility during exocytosis within a single cell (Park et al., 2012; Wilhelm et al., 2014; Kruss et al., 2017). However, its temporal resolution is not enough to determine the kinetic parameters of exocytotic events which could be obtained with SCA (Liu et al., 2017). To maximize the complementary advantages of TIRFM and SCA, a fluorescent false neurotransmitter 102 (FFN102) with both pH-dependent fluorescence and electroactivity was synthesized and used as a dual functional probe to track vesicular exocytosis with high spatial and temporal resolution using a coupled technique (TIRFM and SCA) (Liu et al., 2017). N13 cells, a stable clone of BON cells, were preincubated with FFN102 and then placed on a microdevice containing eight independent indium tin oxide (ITO) electrodes, one of which was set at +900 mV (vs. Ag/AgCl). Stimulation with 10 μM ionomycin, a calcium ionophore, resulted in extinctions of fluorescent vesicles in an individual cell with distinct spatial resolution showing the location of the releasing site and the movement of vesicles along the exocytotic period in TIRFM measurement. An independent SCA experiment recorded a series of current transients, representing the oxidation of FFN102 released on the ITO electrode, at a single N13 cell after simulated with 10 μM ionomycin. Subsequently, TIRFM/SCA-coupled detection was monitored at the same cell successfully. As a typical example, Figure 4 depicted the correlation of four fluorescence images recorded with a time interval of 25 ms and amperometric signal for a single vesicle which released the neurotransmitter at the active zone. With this well-defined correlation, one can localize the release vesicle in a living cell precisely with TIRFM while quantitating the release kinetics of the neurotransmitter from this vesicle with SCA with excellent temporal resolution.

### Ascorbate

**Figure 5:**
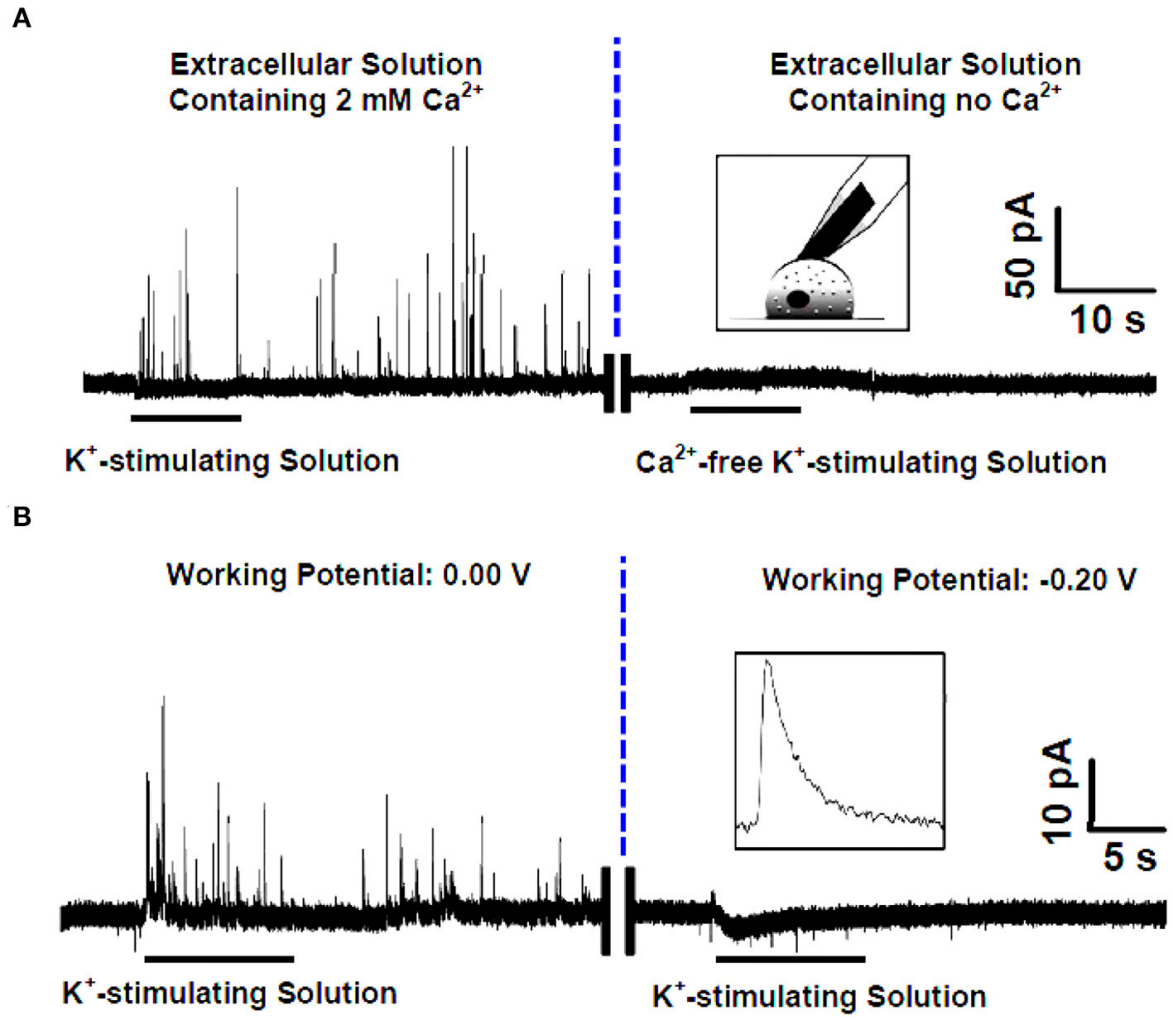
**(A)** (Left) Amperometric spikes recorded with the pretreated carbon fiber microelectrode (CFE) closely attached to a single rat adrenal chromaffin cell that was cultured in the standard extracellular solution (i.e., containing 2 mM Ca^2+^) and stimulated by K^+^-stimulating solution. (Right) Current-time trace recorded with the pretreated CFE closely attached to a single rat adrenal chromaffin cell that was cultured in the Ca^2+^-free extracellular solution and stimulated by Ca^2+^-free K^+^-stimulating solution. Inset, schematic illustration of the single-cell amperometry with the pretreated CFE. **(B)** High K^+^ evoked amperometric spikes recorded with the pretreated CFE closely attached to a single rat adrenal chromaffin cell cultured in the standard extracellular solution (i.e., containing 2 mM Ca^2+^) at different holding potentials of 0.0 V (left) and −0.20 V (right). Inset, a typical amperogram spike for ascorbate secretion. Reprinted from Wang et al. (2017), with permission from the American Chemical Society.

Ascorbate, the salt form of vitamin C in physiological conditions, acts as a neuromodulator of glutamatergic, cholinergic, dopaminergic, and GABAergic transmission (Xiao et al., 2018, 2019; Jin et al., 2020; Yu et al., 2020). It has been proposed that one pathway of cellular efflux of ascorbate is through co-secretion with catecholamine during exocytosis in chromaffin cells (Daniels et al., 1982). To investigate whether and how this happens, Wang et al. developed an elegant SCA method to selectively monitor endogenous ascorbate secretion from single chromaffin cells during exocytosis (Wang et al., 2017). To overcome the interference from the dominant neurotransmitters, such as norepinephrine, epinephrine, and DA in chromaffin vesicles, the carbon fiber microelectrodes (CFMEs) were pretreated in 10 mM sodium tetraborate (pH 9.5) at +1.3 V for 20 min. Cyclic voltammetry (CV) showed that after pretreatment, the oxidation potential of ascorbate was decreased to 0.0 V (vs. Ag/AgCl), at which the interference of catecholamines could be ignorable. As shown in Figure 5, high K^+^ elicited many amperometric spikes representing ascorbate secretion from single vesicles during exocytosis in a Ca^2+^-dependent manner.

### Glutamate

Glutamate, the most dominant excitatory neurotransmitter in mammalian CNS, plays key roles in many life functions, such as memory formation, long-term potentiation, and synaptic plasticity (Herman and Jahr, 2007; Abramov et al., 2009; Brito-Moreira et al., 2011). Since the electrochemical activity of glutamate is poor at the regular electrode material, various enzymatic biosensors have been developed to monitor the glutamate concentration and the fluctuation of glutamate in the brain (Wu et al., 2018; Ganesana et al., 2019). Usually, the specific enzyme layer immobilized on the electrode with hydrogels is relatively thick for the purpose of loading enough enzyme for the substrate catalysis, so the diffusion of substrate to the electrode is slow thus leading to slow response speed. This is the key factor that restricts enzymatic biosensors to be applied in monitoring neurotransmitter release in vesicular exocytosis that requires high temporal resolution.

**Figure 6:**
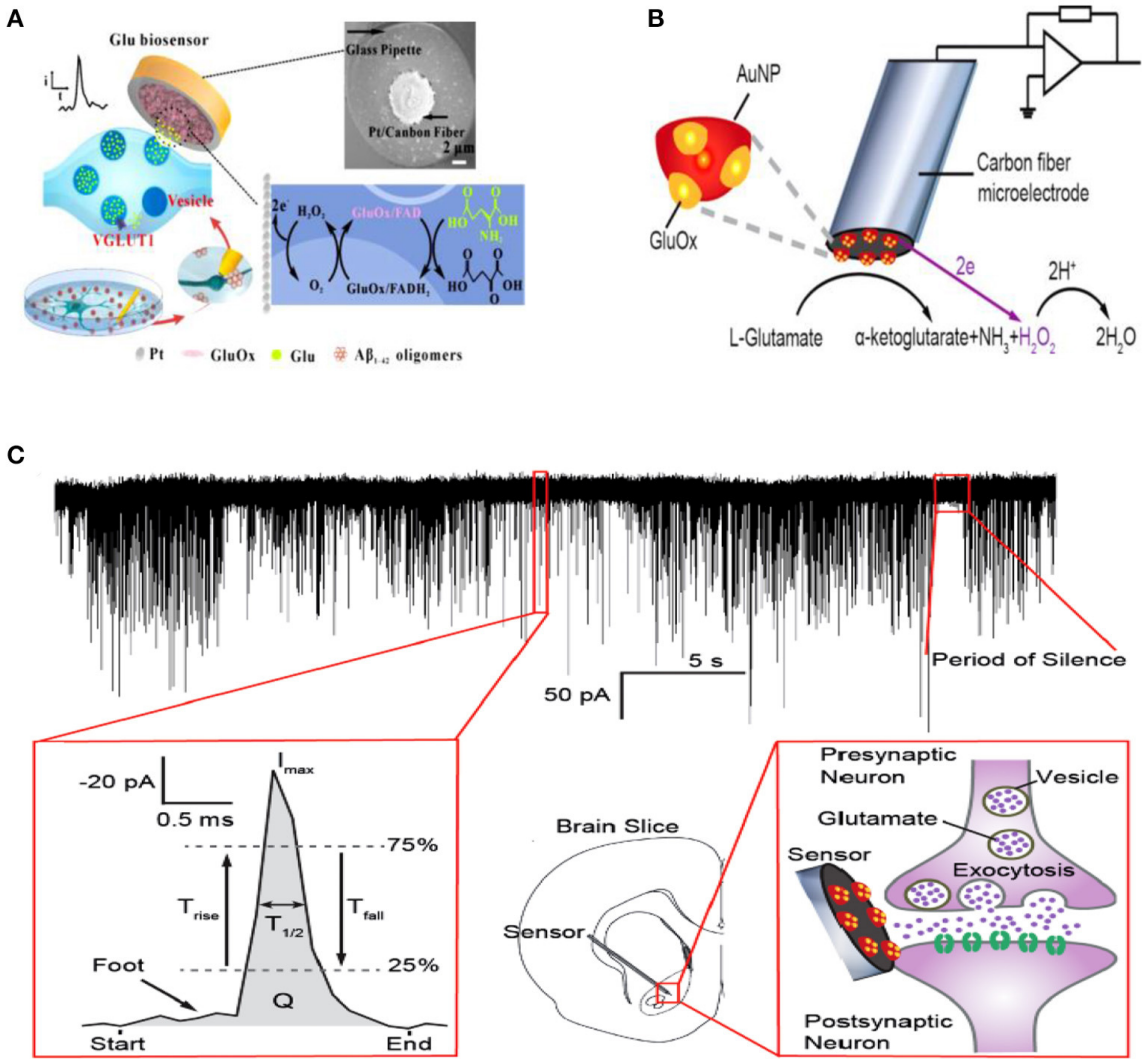
**(A)** Schematic diagram showing the process of amperometric monitoring of glutamate exocytosis from single hippocampal varicosity; the top right shows the scanning electron microscope (SEM) images of a platinized carbon fiber microelectrode, and the bottom right shows the mechanism of Glu detection on the microsensor. **(B)** Schematic diagram of the amperometric glutamate sensor design consisting of a glutamate oxidase (GluOx)-coated gold nanoparticle-modified carbon fiber microelectrode. It displays the chemical enzyme catalysis reaction chain for glutamate with the subsequent detection scheme for electrochemical detection of the reporter molecule H_2_O_2_ produced. The red hemispheres represent gold nanoparticles, and GluOx that is immobilized at the surface is displayed in yellow (not drawn to scale). **(C)** Top: Amperometric current–time trace detecting spontaneous glutamate release during individual exocytotic events in mouse brain slice. Below: Definition of the current spike parameters used for exocytosis kinetic analysis after converting the amperometric recording to positive reduction current (left). Illustration of the placement of sensor in the nucleus accumbens of rodent brain slice for amperometric recording. Schemes are not drawn to scale (right). Reprinted from Yang et al. (2019) and Wang et al. (2019b), with permission from the American Chemical Society.

To accelerate the response speed of glutamate enzymatic biosensor, Huang's group constructed a glutamate biosensor with the following brief steps: (1) platinization of CFME; (2) modification of polyetherimide (PEI) on the surface of platinized CFME; and (3) immobilization of glutamate oxidase (GluOx) through the covalent linking with poly(ethylene glycol) diglycidyl ether (PEDGE) (Qiu et al., 2018). Herein, GluOx catalyzes the oxidation of glutamate to α-ketoglutarate accompanied with the reduction of O_2_ to H_2_O_2_ which could be monitored with platinized CFME effectively (Figure 6A). This assembled sensor exhibited excellent sensitivity and good selectivity, more importantly, fast response speed (response time, 70 ms) to glutamate, resulting in the capability of monitoring the dynamics of glutamate release in exocytosis. The following SCA experiments with this sensor at single hippocampal varicosity showed that many well-defined amperometric spikes were recorded specifically in the presence of GluOx on the electrode when high K^+^ was applied. It was confirmed that this new glutamate sensor is efficient to measure the glutamate release during exocytosis. Using this approach, they further investigated the effect of Aβ_1−42_ oligomer protein, which is proved highly related with Alzheimer's disease (AD), on the level of glutamate release in exocytosis in single hippocampal varicosity (Yang et al., 2019). The results showed that a short-duration (30 min) incubation with Aβ_1−42_ increased vesicular glutamate release dramatically while a long-duration (300 min) treatment depleted vesicular glutamate release. It could provide valuable information on Aβ_1−42_-induced abnormalities in neuron functions and the early pathogenesis of AD.

As another attempt, Wang et al. measured glutamate release during exocytosis through monitoring the reduction of H_2_O_2_ instead of its oxidation on the GluOx-modified microelectrode (Figure 6B) (Wang et al., 2019b). In this approach, gold nanoparticle hemispheres were deposited on CFME (33 μm in diameter) firstly to not only increase the electrode surface area but also provide a high surface curvature to keep the activity of GluOx. Then, an optimized amount of GluOx was adsorbed on the electrode by keeping the electrode in the GluOx solution for 2–3 h at room temperature, aiming to minimize the thickness of enzyme coating and improving the reaction speed of this glutamate sensor. Once the sensor was placed in the nucleus accumbens (NAc) region of the mouse brain, the authors recorded a train of amperometric spikes spontaneously (Figure 6C), which they ascribed to the spontaneous quantal release of vesicular glutamate in the synaptic cleft. However, more control experiments are desired for the purpose of convincing readers how the relatively huge electrode recorded single exocytotic release of a small amount of glutamate happening in the sub-micrometer synaptic cleft at an ultra-fast speed (milliseconds).

## NanoITIES

The exocytotic dynamics of electrochemically active neurotransmitters and neuromodulators, such as DA, norepinephrine, epinephrine, and ascorbic acid, can be revealed by SCA, which provides a platform for studying related diseases. However, non-redox active neurotransmitters, such as acetylcholine (ACh), γ-aminobutyric acid (GABA), and glutamate, are equally important for brain functions. Elucidating the dynamic process of these neurotransmitters in exocytosis is extremely helpful for understanding the pathogenesis of related neurological diseases. While a specific enzyme nanosensor provides one strategy for monitoring non-redox active neurotransmitters, the nanoscale interface between two immiscible electrolyte solutions (nanoITIES) opens another wide window especially for the ionic neurotransmitters in physiological conditions. Technically, a glass pipette with a nanoscale pore at the tip is back-filled with selected organic reagents and then positioned into a biological environment for measurement. Quantitative current signals of neurotransmitters are obtained through recording the ionic current fluctuations caused by neurotransmitter transfer at the liquid–liquid interface (Wang et al., 2010; Amemiya et al., 2013). Hence, by carefully selecting the organic reagent, nanoITIES can specifically measure the concentration and release dynamics of ionic neurotransmitters at the single-cell level with high spatial resolution.

The ion transfer of three functional chemicals, including ACh, serotonin (5-HT), and tryptamine, at the interface of 1,2-dichloroethane (DCE)/artificial seawater (ASW) was studied by using a nanoITIES pipette electrode with a radius of 7–35 nm (Colombo et al., 2015). The difference of the half wave transfer potentials for these three chemicals and the linear response to their concentration confirmed that the nanoITIES electrode could measure these chemicals quantitively and selectively.

GABA, an inhibitory neurotransmitter, was analyzed with nanoITIES (Iwai et al., 2018). Since GABA is electrically neutral in physiological conditions, organic acid was added to the original water/1,2-DCE interface to facilitate the production of a GABA ionic complex, which can be detected once it transfers through the nanopore of ITIES. This lays the foundation for the subsequent direct measurement of GABA at the single-cell level.

**Figure 7:**
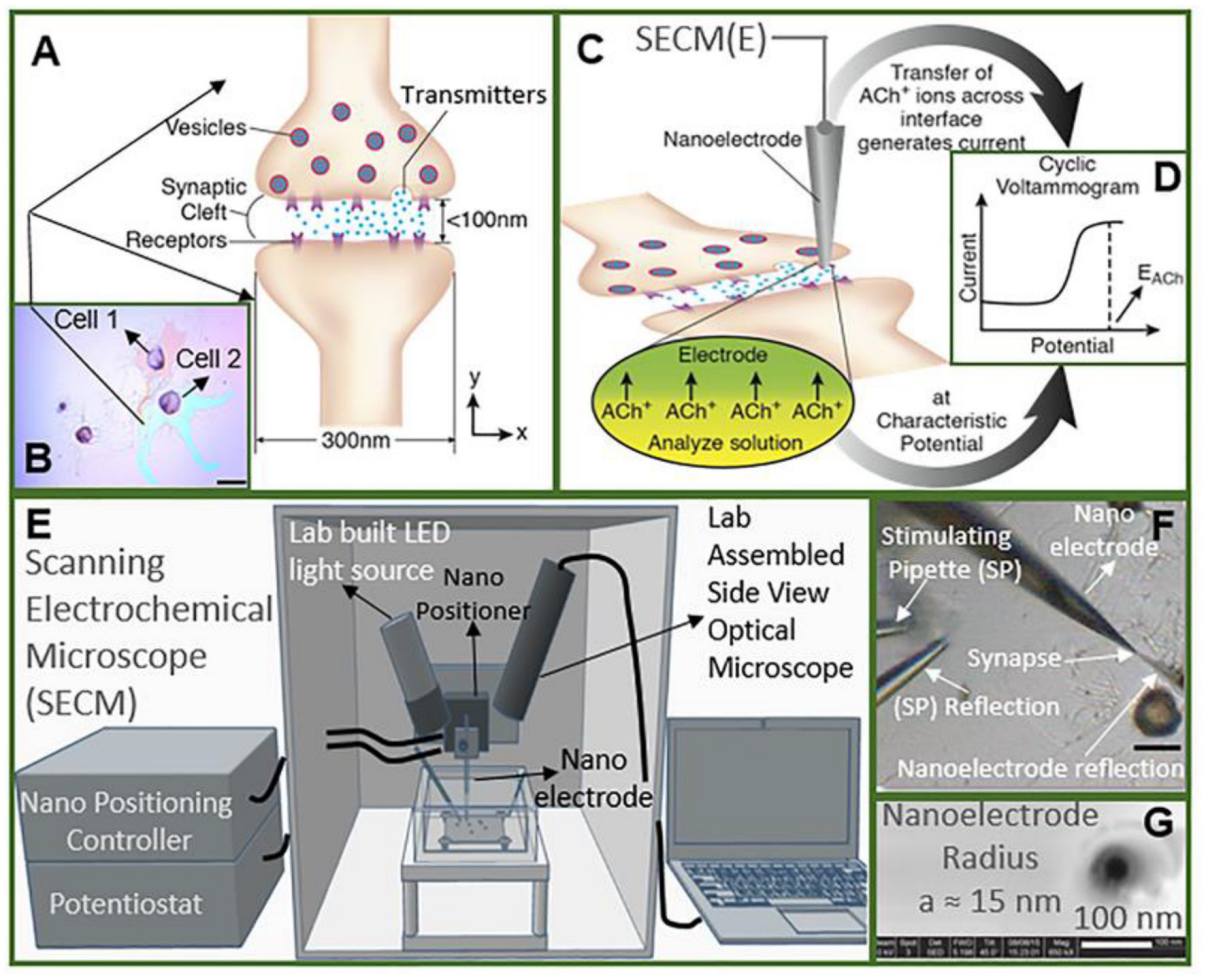
Study of cholinergic neurotransmission at the single synaptic cleft with nanoelectrode and scanning electrochemical microscope (SECM). **(A)** Illustration of synaptic cleft dimensions. **(B)** Cultured living aplysia pedal ganglion neurons used for the experiment, where the axon from cell 1 (pink) formed a synaptic connection with the body of cell 2. Scale bar: 200 μm. **(C)** A nanoscale interface between two immiscible electrolyte solutions (nanoITIES) pipette electrode was positioned around the synaptic cleft to measure the concentration and release dynamics of acetylcholine (ACh^+^) simultaneously using amperometry; the positioning of the nanoelectrode was achieved using the SECM with a spatial resolution of 5 nm. The zoom shows the nanoITIES formed at the tip of the nanoITIES pipette electrode, and ionic transmitter (ACh^+^) transfers across the interface, generating a current and thus getting detected. **(D)** Cyclic voltammogram corresponding to ACh detection, where the detection potential follows the Nernstian equation, and a steady-state transfer potential, E_*ACh*_ = −0.48 V vs. E_1/2_, _*TBA*_, selective for cholinergic neurotransmitter detection was used in amperometry to study its synaptic concentration dynamics. **(E)** A SECM and a lab-built side view optical microscope were used for the positioning of the nanoelectrode around synapses with a nm spatial resolution. The lab-built side-view optical microscope provided rough positioning before the fine positioning of 5 nm spatial resolution with SECM. After SECM positioning, the optical microscopic view of the nanoelectrode and the synapse are shown in **(F)**, where it can be seen that it is very hard to locate the synapse by visual observation alone. The combined use of the side-view optical microscope and nano-positioning platform, SECM, is critical. **(F)** A stimulating pipette was used to provide high-concentration K^+^ stimulation. Reflection was used for the rough positioning of the nanoelectrode and stimulating pipette in the x, y, and z axes by an optical microscope, which was followed by the nanometer positioning of the nanoelectrode around the synapse achieved using nano-resolution SECM with details described in the supporting information. Scale bar: 150 μm. **(G)** High-resolution scanning electron microscope (SEM) picture of the nanopipette tip with the radius to be around 15 nm. Reprinted from Shen et al. (2018), with permission from the American Chemical Society.

Recently, Shen's group monitored the concentration and release kinetics of ACh from an individual neuronal soma and at a real single synapse of *Aplysia nica* with a nanoITIES pipette electrode (Shen et al., 2018; Welle et al., 2018). As shown in Figure 7, the nanoITIES electrode can be accurately positioned near the synaptic cleft assisted by scanning electrochemical microscopy (SECM), an electrochemical technique that employs ultramicroelectrodes to record a faradaic current response while moving in proximity of a sample surface with extreme precision. The current response can reflect both the topography and the electrochemical activity of the surface/substrate (Page et al., 2017; Stephens et al., 2020) and a side optical microscope. Obvious amperometric spikes could be obtained at the nanoITIES pipette electrode for ACh release from the presynaptic membrane under the stimulation of a high concentration of K^+^. They found that ACh release from the *Aplysia* neuron is Ca^2+^ dependent and its dynamic profiles are composed of singlet, doublet, and multiplet. This breakthrough for quantitative monitoring of ACh release in a real synapse opens up new opportunities to understanding neuronal communication and related diseases.

## Using Electrochemical Cytometry to Determine Neurotransmitter Storage of Single Vesicles

Quantitatively monitoring neurotransmitter release with high temporal resolution methods, such as SCA and ITIES, provides important information for chemical signaling between neurons. However, increasing evidence has indicated that neurotransmitter release during exocytosis is not the simple “all or none” (Elhamdani et al., 2001; Sombers et al., 2005; Camacho et al., 2006; Haynes et al., 2007; Omiatek et al., 2010, 2013; Dunevall et al., 2015; Calvo-Gallardo et al., 2016). For instance, Borges's group investigated catecholamine release in a single exocytotic event at single chromaffin cells with SCA and patch amperometry in which physical suction is applied at a small portion of the cell membrane to create sealing for capacitance measurements (Montesinos et al., 2008). Although not the main focus of their study, they found that the total charge released per exocytotic event stimulated with Ba^2+^ was 0.67 ± 0.08 pC measured with SCA, whereas the total charge stimulated with mechanical suction is 1.81 ± 0.09 pC measured with patch amperometry for the same cell line. To address whether and how the amount of neurotransmitter released in exocytosis is controlled, quantitation of neurotransmitter storage in single vesicles is essential. Therefore, a series of advanced electrochemical technologies, all termed as electrochemical cytometry, have been developed.

### Vesicle Impact Electrochemical Cytometry (VIEC)

**Figure 8:**
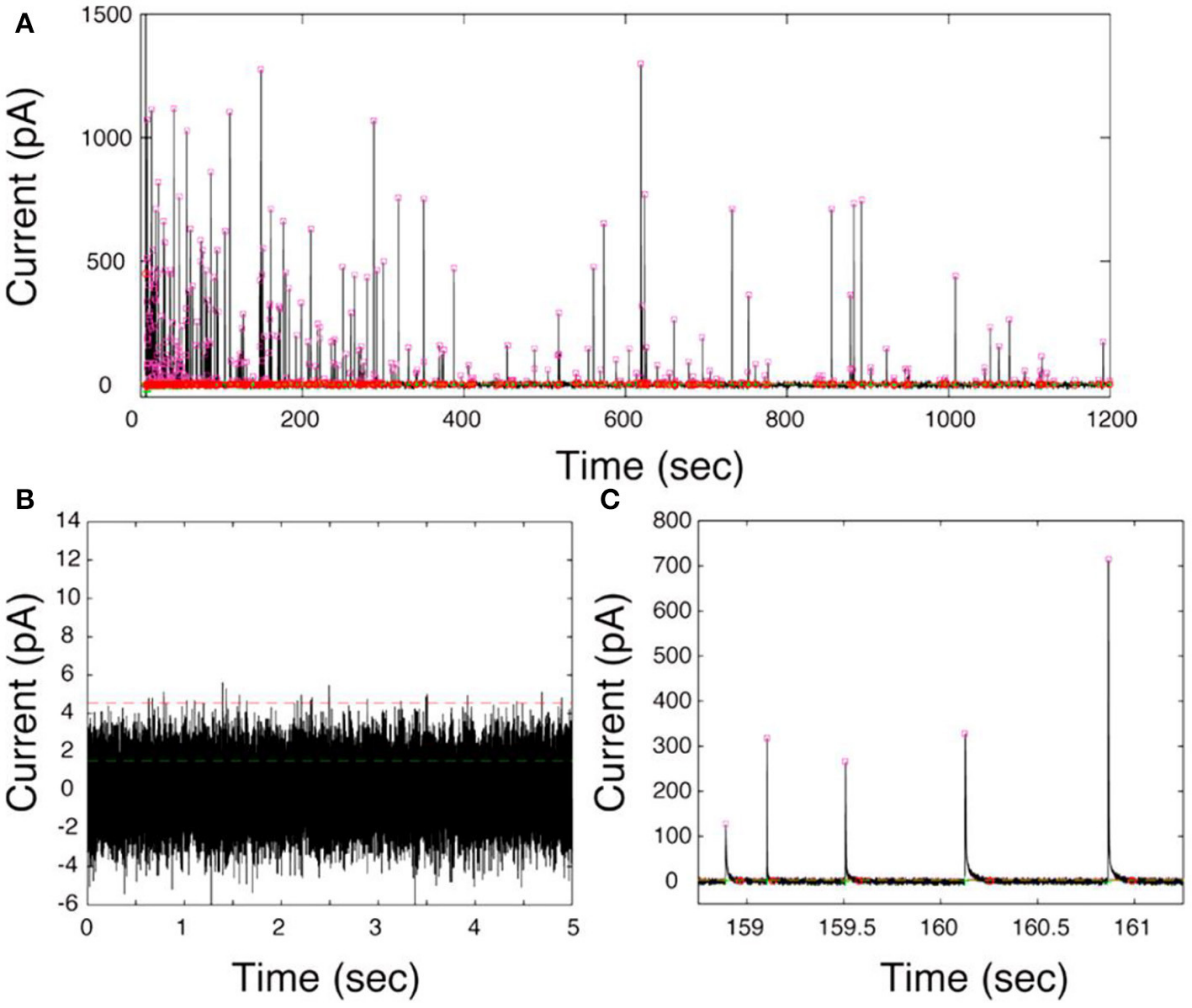
**(A)** Representative current–time trace of VIEC in a suspension of chromaffin cell vesicles. **(B)** A 5-s baseline at 0 mV vs. Ag/AgCl in the presence of vesicles. **(C)** Expanded view of current transients. The pink squares represent the *I*_max_ of all peak candidates submitted for further analysis. The green lines represent the root mean square (RMS) and the red lines five times the RMS of the baseline noise. Reprinted from Dunevall et al. (2015), with permission from the American Chemical Society.

The first generation of electrochemical cytometry (EC) could be traced back to a decade ago. Omiatek et al. coupled capillary electrophoresis with electrochemical detection on a microfluidic device to achieve the quantitative determination of neurotransmitters stored in single mammalian vesicles (Omiatek et al., 2009, 2013). Since the complicated device limits its application, further attempts have been performed on liposomes [i.e., artificial vesicle, with the inspiration of single-entity electrochemistry to build a simpler system (Cheng and Compton, 2014; Kim et al., 2014)]. In 2015, the second generation of EC, termed as vesicle impact electrochemical cytometry (VIEC), was successfully developed (Dunevall et al., 2015). In this system, the CFME recorded bunches of oxidized current transients once it was put into a concentrated vesicle suspension isolated from adrenal chromaffin cells and a potential at + 0.7 V vs. Ag/AgCl applied (Figure 8). Quartz crystal microbalance experiments provided evidence for vesicle adsorption and rupture during VIEC. Through data processing, the estimated diameters of the vesicles calculated with the number of molecules measured in VIEC matched very well with the direct measurement with nanosight tracking analysis. This suggested that the current transients recorded in VIEC were generated from the oxidation of catecholamine stored in single vesicles.

**Figure 9:**
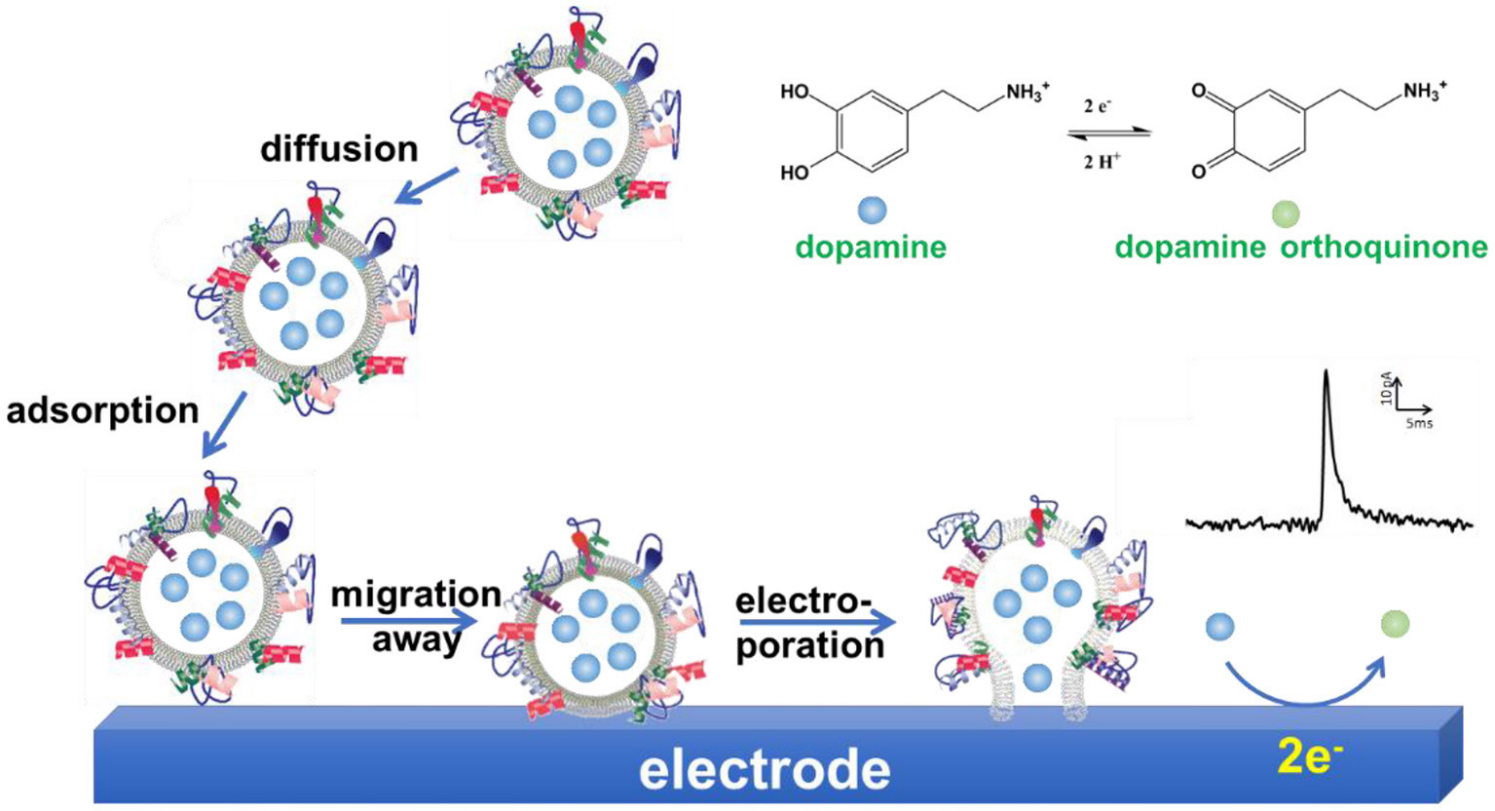
Schematic illustration of a proposed mechanism of vesicle impact electrochemical cytometry (VIEC).

Further studies were performed to explore the mechanism of VIEC (Lovric et al., 2016; Li X. et al., 2017; Phan et al., 2017; Li et al., 2018). Mathematics simulation confirmed that the amount of neurotransmitters measured with VIEC was the total content in single vesicles, suggesting all the vesicular transmitters were expelled and oxidized on CFME (Li et al., 2018). Gradually increasing the applied potential on the working electrode increases the frequency of current transients gradually when other conditions were kept constant (Lovric et al., 2016). Analysis of the current transients indicates that a nanoscale pore was formed on the vesicle at the initial state (Li X. et al., 2017). Therefore, electroporation was proposed to be involved in VIEC. Moreover, the comparison of mammalian vesicles, lipidic liposomes decorated with a small amount of proteins, and pure lipidic liposomes in VIEC showed that the frequency of detected current transients was in an order F_mammalianvesicle_ < F_protein−decoratedliposome_ < F_pureliposome_ (Lovric et al., 2016). Subsequent studies demonstrated that the increase of detection temperature could promote the occurrence of electroporation and increase the frequency of vesicular rupture events through increasing the diffusion rate of membrane proteins (Li X. et al., 2017). These two studies imply that vesicular membrane proteins affect the occurrence of electroporation, thus playing an important role in vesicle rupture on electrodes. Taking all together, a mechanism was proposed for quantification of vesicular catecholamine content with VIEC (Li X. et al., 2016a; Phan et al., 2017). As shown in Figure 9, vesicles were adsorbed on the working electrode firstly. Then the vesicular membrane protein in the proximity of the electrode migrates away, resulting in the gap between vesicle membrane and electrode close enough to facilitate electroporation. Through the nanosized pore formed, neurotransmitters fluxed to the electrode and were oxidized to produce a current transient on the electrode surface, making the quantification of neurotransmitter storage in single vesicles practical (Li X. et al., 2016a; Phan et al., 2017; Ranjbari et al., 2019).

In a recent report, the enzymatic glutamate sensor schemed in Figure 6B was applied to determine the glutamate storage in individual isolated synaptic vesicles (Wang et al., 2019a) with the same manner of VIEC. For reasons they did not state, a calibration curve obtained with large unilamellar vesicles (LUVs) loaded with a known concentration of glutamate was used to precisely quantify the glutamate amount in individual vesicles instead of the absolute quantification with Faraday's equation. Nevertheless, each isolated synaptic vesicle from the mouse brain was found to contain 8,300 ± 600 glutamate molecules on average. This work provides a new methodology to determine the quantity of glutamate in a single isolated synaptic vesicle, which is helpful to obtain intuitive information of glutamate in neurotransmission.

### Intracellular Vesicle Impact Electrochemical Cytometry (IVIEC)

**Figure 10:**
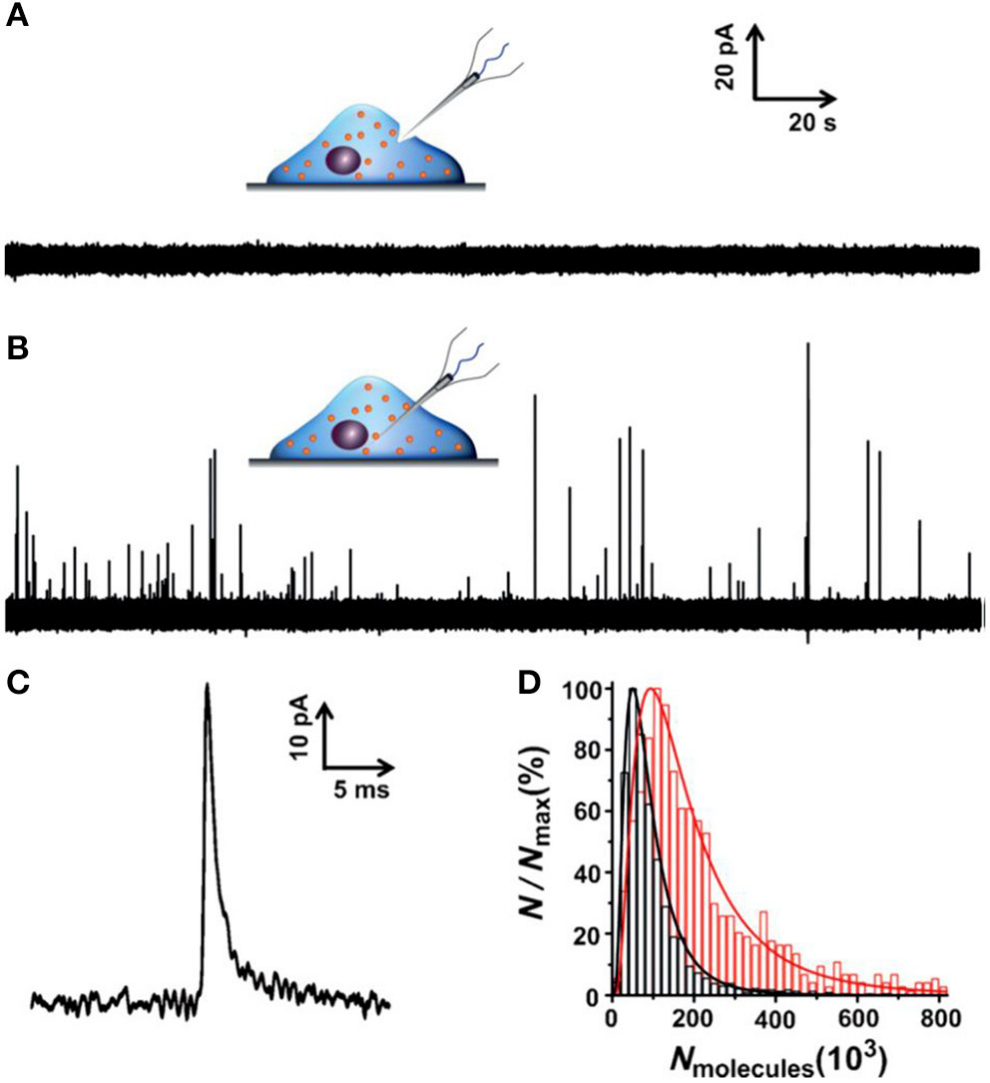
**(A,B)** Amperometric traces for a nanotip conical carbon-fiber microelectrode pushed against a pheochromocytoma (PC12) cell without breaking into the cytoplasm **(A)** or placed inside a PC12 cell **(B)**. **(C)** Amplified amperometric current trace. **(D)** Normalized frequency histograms describing the distribution of the vesicular catecholamine amount as quantified from untreated PC12 cells by intracellular vesicle electrochemical cytometry (red, *n* = 1,017 events from 17 cells) and by K^+^-stimulated exocytosis at the same electrode (black, *n* = 1,128 events from 17 cells). Bin size: 2 × 10^4^ molecules. Fits were obtained from a log-normal distribution of the data. Reprinted from Li X. et al. (2015), with permission from Wiley Online Library.

To minimize the effect of separation processes on vesicle properties, for example, the neurotransmitter leakage during sample preparation, quantification of vesicular content in the native cellular environment is preferred. To achieve this, an elegant and reliable technique, termed as intracellular vesicle impact electrochemical cytometry (IVIEC), was developed using nano-tip conical carbon fiber microelectrodes to quantify the neurotransmitter content of individual vesicles in living PC12 cells (Li X. et al., 2015, 2016a). In this approach, the nano-tip cone geometry readily facilitated the penetration through the cell membrane with little damage to cells and kept enough electrode surface area for the electrochemical reaction of the vesicular neurotransmitter. As displayed in Figure 10, obvious current transients were observed once the electrode was placed inside the cytoplasm of single living PC12 cells while none for the electrode kept outside of the cell. Early reports also observed similar current transients at the carbon fiber electrode when a small portion of the cell membrane was ruptured by suction in whole-cell patch amperometry, and vesicles could diffuse to the electrode, although the authors did not explore these current transients since they focused on quantifying free catecholamine in the cytosol and/or the release of catecholamine from chromaffin cells (Mosharov et al., 2003; Montesinos et al., 2008). Quantification of the single spikes with Faraday's equation revealed that the total amount of vesicular catecholamine storage measured by IVIEC was 114,500 ± 15,300 (mean ± SEM) molecules, which closely agreed with the value measured for freshly extracted vesicles in VIEC (Li et al., 2018). Furthermore, a 2-h treatment of PC12 cells with 100 μM L-3,4-dihydroxyphenylalanine (L-DOPA), a direct biochemical precursor to DA, increased the vesicular catecholamine content to 323,100 ± 29,000 (mean ± SEM) molecules as determined in IVIEC. Both results suggested that the current transients measured in IVIEC were from the oxidation of catecholamine stored in single vesicles, and the mechanism for VIEC is also applicable for IVIEC.

In addition to flame etching used for preparation of nano-tip conical carbon fiber microelectrodes in IVIEC, a wet-etching approach was used to prepare nanoscale sharp-tip nanoelectrodes for investigation of single vesicles in the intracellular environment recently (Roberts et al., 2020). Briefly, a regular cylindrical carbon fiber microelectrode was immersed in 4 M KOH solution and a potential of + 7 V applied (vs. a platinum wire) for 1–1.5 s to cleave a sharp tip. Then, an insulation layer was electropolymerized on the whole electrode except its tip (~5 μm) which was hid in a silicone mask. Once the electrode tip penetrated through the cell membrane and gained access to the cytosol of an individual PC12 cell, a series of amperometric spikes were recorded when the potential was held at + 850 mV vs. Ag/AgCl. The researchers calculated the area of each peak and obtained an average of 140,000 ± 17,000 catecholamine molecules for 35 individual vesicles. Besides, this nanoscale electrode was further used to distinguish epinephrine-containing and norepinephrine-containing vesicles with fast-scan cyclic voltammetry.

### Other Mimic-IVIEC Methods

A similar phenomenon was observed in other methodologies developed for monitoring intracellular reactive oxygen species (ROS) and reactive nitrogen species (RNS). While a platinized carbon nanoelectrode (i.d. 40 nm) was successfully used as SECM tip to measure the dynamic changes of ROS/RNS in the cytoplasm of a variety of human breast cells, a series of amperometric bursts, similar to amperometric events IVIEC, were observed 25 min after the MCF-10A cell was treated with diacylglycerol-lactone (DAG-lactone), a membrane-permeable analog of DAG that induces activation of some kinds of protein kinase C isoforms (Li Y. et al., 2017). Optical imaging confirmed that DAG-lactone treatment induced formation of numerous intracellular vacuoles, a nanoscale membrane compartment. The correlation of amperometric bursts and vacuole formation produced one possibility that vacuoles containing a high concentration of ROS/RNS burst on the electrode and the oxidation of ROS/RNS was detected as spikes, though other reasons could not be ruled out.

In another study, a new platinized nanowire SiC^@^C electrode (Pt-NWES) was developed to quantify ROS/RNS content in single phagolysosomes in living macrophages (Zhang et al., 2017). Similar to IVIEC, a constant potential (+ 0.85 V vs. Ag/AgCl reference) was applied on the electrode, resulting in large series of transient current spikes, each of which corresponds to the oxidation of ROS/RNS stored in single phagolysosomes on the electrode. In their subsequent report, further analysis of the single amperometric spikes showed that 75% events had a clear shoulder on the decay part. They proved that this shoulder was the oxidation of ROS/RNS newly produced inside phagolysosomes because of the stimulation by consumption of ROS/RNS on the electrode (Zhang et al., 2019). These new reports expand the application of IVIEC in cell biology.

### Combining IVIEC With SCA

**Figure 11:**
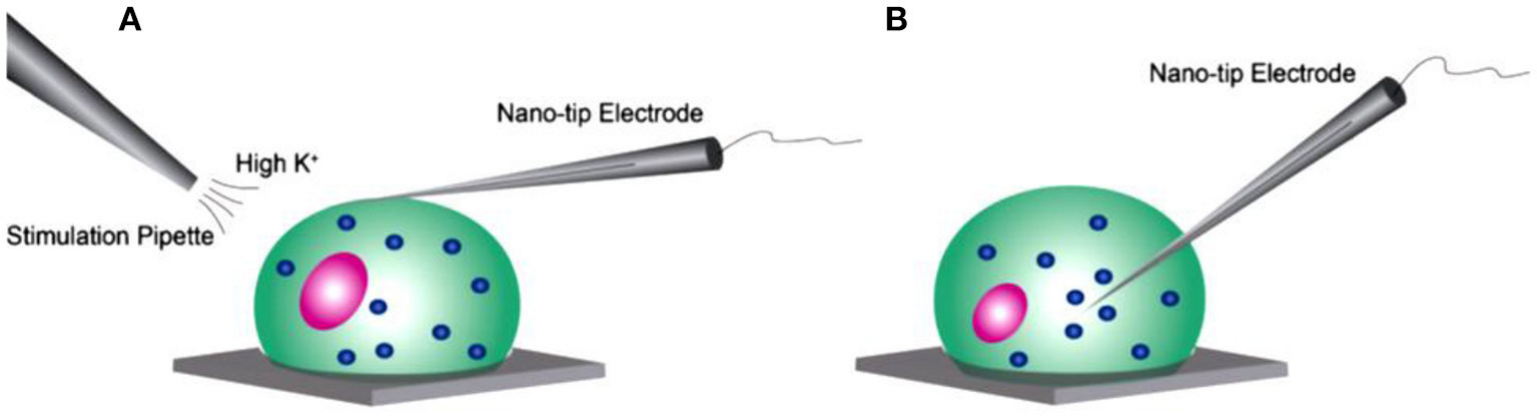
Schematic representation of **(A)** single-cell amperometry (SCA) and **(B)** intracellular vesicle impact electrochemical cytometry (IVIEC). Reprinted from Ren et al. (2017), with permission from Wiley Online Library.

IVIEC can quantify the neurotransmitter storage precisely in individual vesicles in living cells while SCA is capable of quantitatively monitoring the dynamics of neurotransmitter release in exocytosis with very high temporal resolution (Figure 11). Combining these two elegant methods allows researchers to obtain more information on the nature of exocytosis and how related drugs affect its process (Li X. et al., 2015, 2016c; Lovric et al., 2016; Najafinobar et al., 2016; Fathali et al., 2017; Ren et al., 2017, 2019; Ye et al., 2018; Gu et al., 2019; Majdi et al., 2019; Taleat et al., 2019; Zhu et al., 2019; He and Ewing, 2020; Larsson et al., 2020).

#### Exocytotic Mode

The first attempt of combination of IVIEC and SCA was used to understand the exocytosis manner (Li X. et al., 2015). By comparing the catecholamine molecules released determined with SCA and those stored in single vesicles determined with IVIEC, researchers found that an average of ~70% of vesicular neurotransmitters in storage were released during high K^+^-stimulated exocytosis of PC12 cells, suggesting that partial or “subquantal” release is the dominant mode of exocytosis. Treatment of PC12 cells with L-DOPA resulted in the increase of neurotransmitter release and storage with almost equivalent levels, thus keeping the percentage released to be similar to that for control.

Recently, IVIEC was adapted to determine the neurotransmitter catecholamine in single vesicles in a living *Drosophila* larval neuromuscular neuron by inserting a sharp nanotip electrode into a varicosity with the assistance of fluorescence (Larsson et al., 2020). An average value of 441,000 octopamine molecules in storage was obtained for each vesicle. This is much higher than those released in exocytosis determined with SCA. By comparison, ~4.5 and ~10.7% of vesicular octopamine are released for single and complex exocytotic events, respectively. This might suggest that the presynaptic plasticity could be regulated in a huge range (Gu et al., 2019; Zhu et al., 2019).

#### Physical Treatment

The effect of physical treatment on chemical neurotransmission has been investigated with IVIEC and SCA. Gu et al. studied the effects of repetitive stimulation on plasticity of exocytosis and the related mechanism (Gu et al., 2019). The stimuli were performed with a 5-s delivery of 100 mM K^+^ and repeated six times with a 2-min rest interval. In the aspect of exocytosis, the frequency of exocytotic events decreased gradually during short-interval repetitive stimuli, probably due to the decrease of the readily releasable pool (RRP) or fused vesicles. Further analysis of single exocytotic events showed that the molecule number of released neurotransmitters gradually increased during the repetitive stimuli, since a more stable fusion pore was formed during the later stimuli. Although vesicular neurotransmitter storage was slightly reduced after repetitive stimuli, the average release fraction/percentage of neurotransmitter was increased. This is an interesting finding which may provide a direct link between the fraction of neurotransmitter release and exocytotic plasticity.

Early studies have shown that external high osmotic pressure can decrease the quantity of neurotransmitters released during exocytosis (Borges et al., 1997; Troyer and Wightman, 2002). Consistent with these results, Fathali et al. found that the amount of catecholamine released from vesicles in hypertonic solution was greatly reduced compared to that in isotonic solution (Fathali et al., 2017). Moreover, to better understand whether osmotic stress affects the catecholamine content in single secretory vesicles, they measured the vesicular content with IVIEC and found that extracellular hyperosmotic shock (730 mOsm/kg) reduced the vesicular catecholamine content to ~ 60% of its original amount in the isotonic solution. Further characterization with transmission electron microscope (TEM) imaging revealed the vesicle size shrank, mainly in the halo, after cells were treated in the hypertonic environment. Taken together, the results showed vesicles could behave as an osmometer and maintain a relatively constant neurotransmitter concentration.

#### Endogenous Substances

Endogenous substances, such as ions, proteins, and lipids, have been shown to be involved in chemical neurotransmission directly or indirectly with fluorescence imaging or SCA measurement (He and Ewing, 2020; Xin and Wightman, 1998; Lovric et al., 2016; Najafinobar et al., 2016). The role of zinc, an important ion participating in various biological functions, for instance, learning and memory, on chemical neurotransmission was investigated by combining IVIEC and SCA systematically (Ren et al., 2017, 2019, 2020). It seemed that zinc treatment in the range of 0.1–100 μM reduced the vesicular neurotransmitter content, but the amount released during exocytosis was kept constant. The release fraction of neurotransmitters exhibited a clear increasing trend with the increase of zinc concentration, suggesting the function of zinc on the presynaptic strength which might be directly related to learning and memory.

As an essential energy source of organisms, adenosine triphosphate (ATP) is existing in almost all secretory vesicles with a high concentration (Borges, 2013). The study of extracellular ATP treatment using SCA and IVIEC indicated that it increased the release fraction of neurotransmitter during exocytosis, mainly through enhancing exocytotic release since the vesicular neurotransmitter storage was not statistically influenced. Further pharmacological treatment suggested that purinergic autoreceptors might be involved in this process (Majdi et al., 2019).

Recently, the role of DJ-1 protein, an endogenous protein related to Parkinson's diseases (PD), on vesicular storage and release of neurotransmitter was studied with IVIEC and SCA (Yue et al., 2020). DJ-1 deficiency did not alter vesicular catecholamine storage significantly, but it extended the duration and decay time of exocytotic release, which might alter the postsynaptic signaling. This report provided a new insight in understanding the pathogenesis of DJ-1 deficiency-induced PD.

#### Drugs

Cocaine and methylphenidate (MPH) can both inhibit dopamine transporter (DAT), a presynaptic membrane-spanning protein pumping the released DA back into cells, and consequently they both block the reuptake of DA. However, the clearance of DA in the brain by MPH is slower than cocaine, so it is a potential pharmacotherapeutic candidate for treatment of cocaine abuse. Applying SCA and IVIEC at cocaine- and MPH-treated PC12 cells, the results showed that both drugs could reduce the amount of DA released during exocytosis and its storage in single vesicles. However, the fractions of DA released from vesicles went through opposite directions: treatment with cocaine decreased the fraction of neurotransmitter released to 65% when compared with the control (74%), while MPH increased it to 83%. This revealed that besides their inhibition on DAT, other mechanisms might be involved in their effect in chemical signaling (Zhu et al., 2019).

Two typical drugs, lidocaine and barbiturate, were used to demonstrate the effect of anesthetics on chemical signaling using PC12 cells as model cells (Ye and Ewing, 2018). Both drugs exhibit no change on vesicular neurotransmitter content. However, they regulate neurotransmitter release in different ways. Barbiturate selectively closes the larger fusion pores, thus reducing the quantity of catecholamine released during single exocytotic events. Lidocaine modulates the quantity of catecholamine released in a concentration-dependent manner (i.e., high concentration of lidocaine reduces it whereas low concentration enhances it).

The influences of cisplatin and tamoxifen, two anticancer drugs working through different mechanisms, on neuronal chemical transmission, were examined regarding their side effect on memory and cognition [i.e., “chemo-brain” (Li X. et al., 2016c; Taleat et al., 2019)]. Cisplatin affects exocytosis in a dose-dependent manner. Low concentrations of cisplatin stimulate catecholamine release whereas high concentrations inhibit it. Tamoxifen shows a similar function on exocytosis as cisplatin. However, tamoxifen alters the vesicular catecholamine content in a dose-dependent manner while cisplatin shows a subtle effect on vesicular catecholamine storage.

### Combing IVIEC/VIEC With Nano Secondary Ion Mass Spectrometry

**Figure 12:**
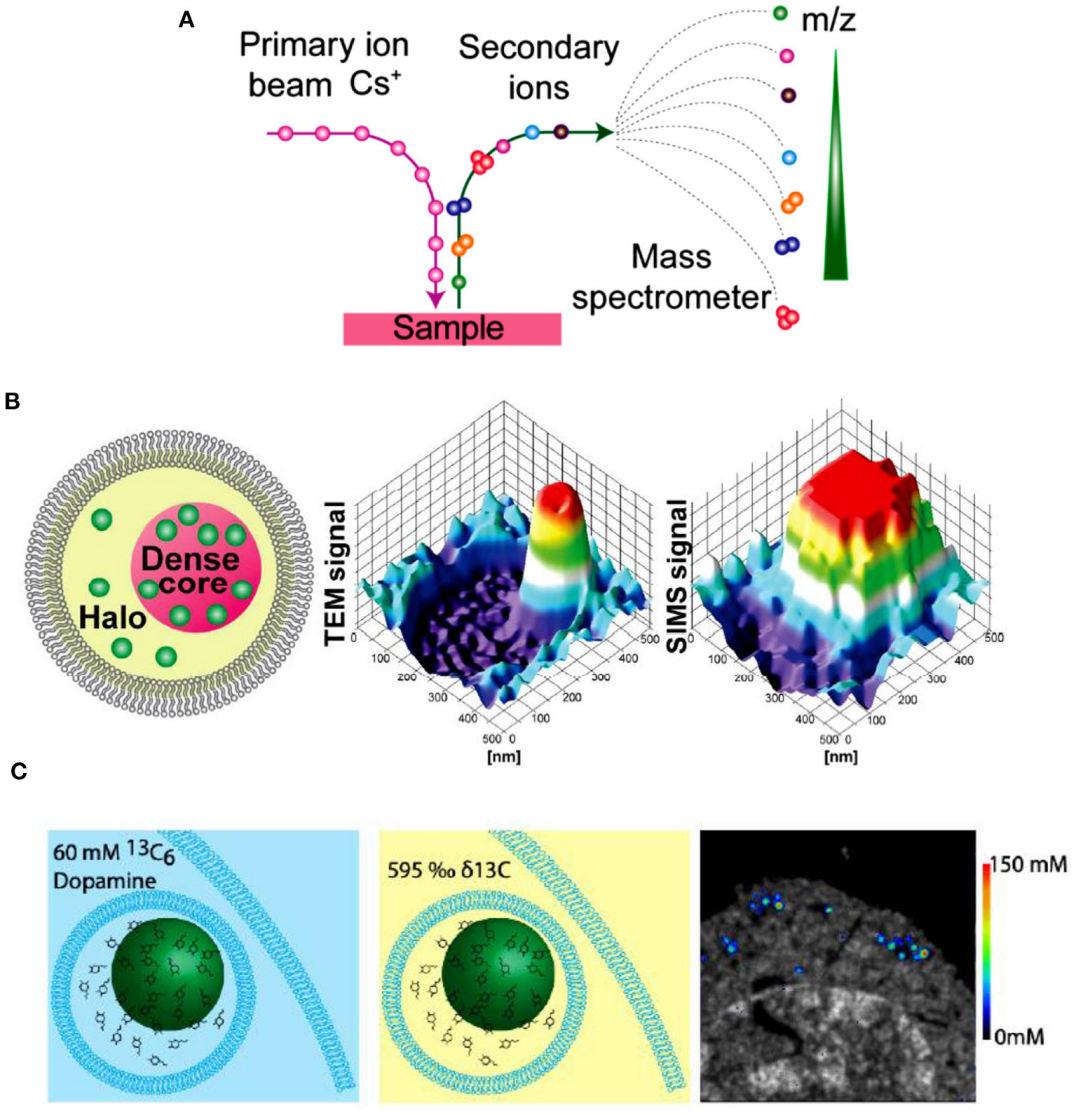
**(A)** Principle of nano secondary ion mass spectrometry (NanoSIMS) measurement. **(B)** Correlation of transmission electron microscopy (TEM) and NanoSIMS imaging to study the distribution of dopamine loading inside single vesicles of PC12 by treatment with ^13^C-L-DOPA and reserpine. From left to right: schematic of a dense core vesicle; 3D surface plots of TEM signals and NanoSIMS signal of ^13^C^14^N. **(C)** The basic concept of a cell pellet embedded in epoxy resin and SIMS concentration imaging. Reprinted from Lovric et al. (2017), Thomen et al. (2020), with permission from the American Chemical Society.

IVIEC has solved the difficulty of precise quantification of neurotransmitter storage in individual mammalian vesicles (ca. 70~200 nm in diameter). Nevertheless, it cannot provide suborganellar neurotransmitter distribution in single vesicles since its spatial resolution can hardly meet this requirement to date. Nano secondary ion mass spectrometry (Nano-SIMS), one of MSI, exhibits elegant capacity to image elements and small chemical fragments at a super high spatial resolution, ~ 50 nm. As schemed in Figure 12A, the impact of high-energy primary ion beam on the sample surface produces secondary ions, which enter the magnetic sector mass analyzer subsequently and are recorded as the *m/z* of the ions. Then, we can obtain the chemical composition of the sample surface. Moreover, the two-dimensional or three-dimensional chemical imaging of the sample can be obtained by scanning and sputtering the sample surface with Nano-SIMS.

In an effort to investigate the mechanism of neurotransmitter storage in single mammalian vesicles, Lovric et al. combined two imaging techniques (Nano-SIMS and TEM) with IVIEC to analyze the distribution of DA across single nanoscale vesicles in PC12 cells, as shown in Figure 12B (Lovric et al., 2017). Correlation between imaging and electrochemical data demonstrates that the transfer of DA between vesicular compartments, dense core and halo, is slow and kinetically limited. More recently, in order to achieve the absolute quantification of the concentrations of metabolites and drugs at the nanoscale level across organelles, Thomen et al. developed a Nano-SIMS method for absolute quantification of DA in single vesicles extracted from PC12 cells. In this work, VIEC was used to validate the vesicular DA content determined with Nano-SIMS. Both methods showed that the concentration of DA in PC12 vesicles is about 60 mM, crossly validating the reliability of these methods (Thomen et al., 2020).

### Combing VIEC With Resistive-Pulse Sensing

Resistive-pulse sensing is a very sensitive electrochemical method which can study the properties of single entities, such as nanoparticle and macromolecule. The principle of this technique is to monitor the passage and translocation of the target by recording the ionic charge transfer at the tiny micro/nanopores when the target is passing through it (Holden et al., 2011, 2012; Li et al., 2016; McKelvey et al., 2016; Yu et al., 2019). Resistive pulse sensors have been developed and used to study the properties of individual artificial vesicles, such as size and surface charge (Chen et al., 2015; Darvish et al., 2016; Liu et al., 2018; Pan et al., 2019, 2020).

**Figure 13:**
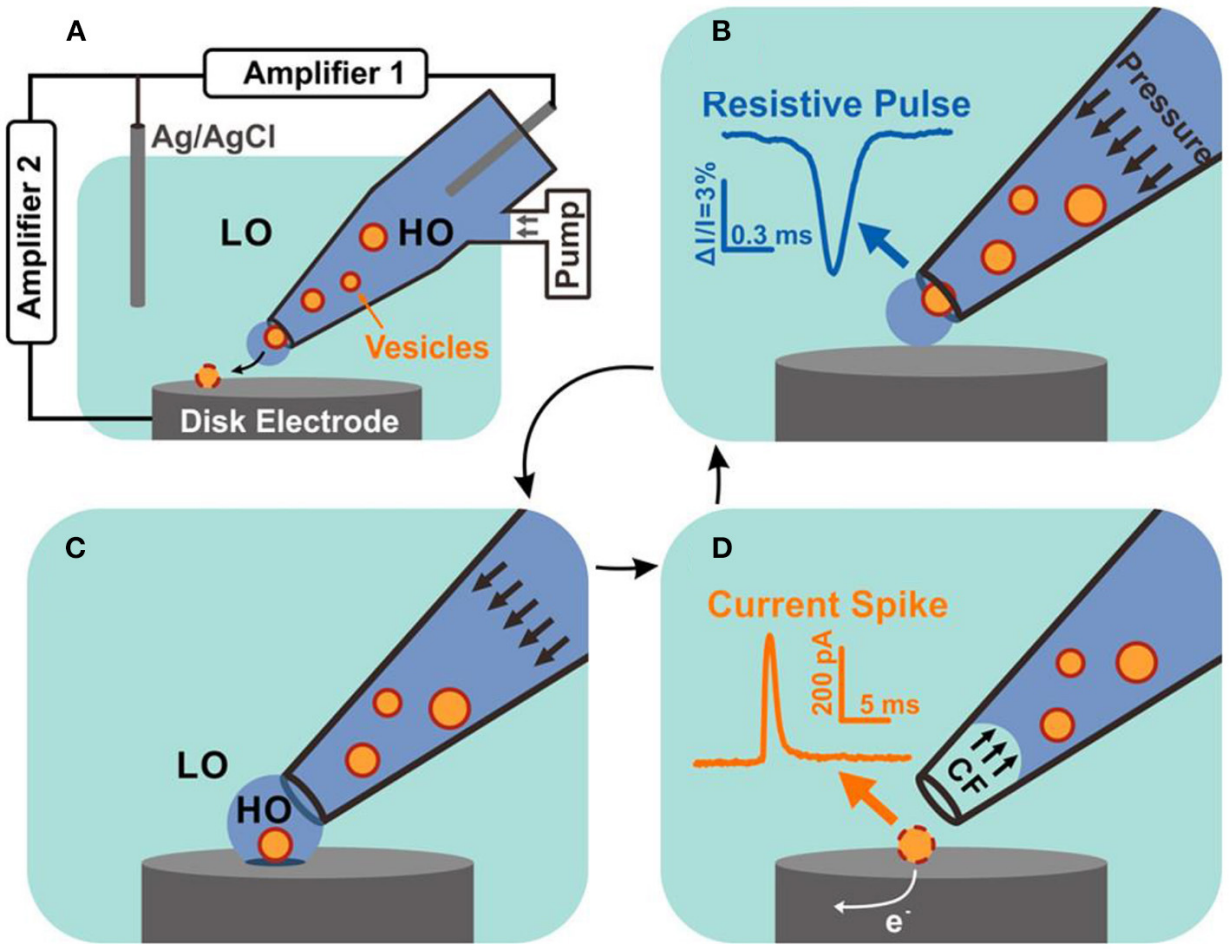
Schematic diagram of resistive pulse-vesicle impact electrochemical cytometry (RP-VIEC). **(A)** Electrode configuration for RP-VIEC. Amplifier 1 records the RP at a potential of +13 mV vs. Ag/AgCl reference electrode. Amplifier 2 records the current spike for VIEC with electrode potential set to +700 mV vs. the same reference electrode. **(B–D)** Schematics showing a cycle induced by periodic pressure: **(B)** Pressure is applied to push a vesicle across the nanopore and generate an RP signal. **(C)** The vesicle attaches on the electrode surface and is surrounded by the outflowing buffer with relatively high osmolality (similar to vesicular lumen). LO, low osmolarity; HO, high osmolality. **(D)** Suspended pressure results in capillary force (CF) stopping solution outflow. The vesicle on the surface opens by electroporation aided by the relatively low osmolarity of the surrounding solution. Electroactive content of the vesicle is electrooxidized and generates a current spike. Reprinted from Zhang et al. (2020), with permission from the American Chemical Society.

In a recent report, Zhang et al. combined the resistive pulse sensing with VIEC to simultaneously quantify the size and neurotransmitter content of individual nanoscale vesicles extracted from bovine adrenal glands (Zhang et al., 2020). As shown in Figure 13, single vesicles initially placed in the glass pipette passing through the nanopore powered by a periodic pressure produce a resistive pulse which could reflect the vesicle size quantitatively. Once the vesicle comes out of the pipette and reaches the disk microelectrode, the vesicle content is expelled which is powered with electroporation with extra assistance of surfactant and electrochemically oxidized on the electrode. Then, vesicle content could be evaluated by analyzing the produced current spike. They successfully quantized 278 vesicles with this combined technique and found out the vesicular catecholamine concentration differed in dense core vesicles and non-dense core vesicle.

## Conclusion and Perspectives

Electrochemistry assembled with micro/nano electrodes provides a powerful tool for quantitative monitoring of neurotransmitter released from or stored in single vesicles. The exocytosis of several kinds of neurotransmitter or neuromodulator, such as catecholamines, ascorbate, glutamate, and Ach, has been successfully examined with electrochemical methods, including SCA and ITIES. The development of VIEC and IVIEC opens a new era for studying the vesicular content which may influence exocytosis. Combination of VIEC/IVIEC and SCA/Nano-SIMS/resistive-pulse sensing enables the quantitative investigation of single vesicles from multidimensions, including chemical contents storage, distribution, release, and physical properties.

Despite the elegant temporal resolution as well as its great spatial resolution and high sensitivity which promote the development of electrochemical approaches and their breakthrough application in neuroscience study, there are still unmet challenges. First, most amperometric studies are still limited for electrochemically active neurotransmitters, for example, catecholamines; the amperometric methodology of quantitatively examining the release of other kinds of neurotransmitters, such as ACh, neuropeptides, nucleotides, and nitric oxide is still highly desired though several attempts have been carried out. The enzyme-based electrochemical sensors and ITIES have great potential in this field although they have their own disadvantages to overcome. Secondly, improving the spatial resolution of electrochemical methods and imaging the neurotransmitter release during exocytosis at the nanoscale level is another direction to strive. In addition, distinguishing several kinds of neurotransmitters which exist in the same location brings another challenge for electrochemical methods. The interdisciplinary aspects which can bring new knowledge across fields may create new opportunities for meeting those challenges.

## Author Contributions

All authors listed have made a substantial, direct and intellectual contribution to the work, and approved it for publication.

## Conflict of Interest

The authors declare that the research was conducted in the absence of any commercial or financial relationships that could be construed as a potential conflict of interest.
